# Notes of a Trip around the World

**Published:** 1913-05

**Authors:** Regina Flood Keyes

**Affiliations:** Buffalo, N. Y.


### Notes of a Trip Around the World

BY DR. REGINA FLOOD KEYES
Buffalo, N. Y.

THIS trip was made on the Hamburg-American Liner Cleveland, starting from New York October 19, 1912, and returning to San Francisco in January, 1913. About 400 passengers were carried and a crew of 375. Beyond sea sickness and a few minor ailments the only medical cases occurring were two, each in a man aged 76, one of whom dropped dead of apoplexy and the other died of perforating cancer of the intestine. The general good health was due to the extreme vigilance of the officers. Only boiled water was drunk and food was purchased with the greatest care. In Java and China the passengers were not even allowed to buy fruit, on account of the custom of using human manure on the fields, and a special supply of water was sent with passengers on land trips, even on trains.

At Manila the company found the best hotel outside of the U. S. and the only closed sewers. Two old Buffalo boys were met in Manila, Dr. Ernest Bingham, stationed at Fort McKinley, who expects to return to the States within a year; and Dr. A. B. Goff, in charge of St. Lazare Infectious Hospital. This hospital is thoroughly up-to-date. Aside from 250 lepers, there was only one patient in the hospital at the time of the visit, this one having varioloid. Dr. Goff has been in the Philippine Islands for 12 years, and is a member of the Manila Board of Health.

Capt. Wm. Davis, M. D., who left Fort Porter in November, 1912, is at Fort Scofield, in Honolulu, with 4000 troops under his medical supervision.

The most interesting medical observation was of the preparation of antivenoms (sic) at Bombay. Two venoms are used, those from the cobra and Russell's viper, either antivenom protecting against either venom, indiscriminately, although bivalent antivenom has also been prepared. The snakes are kept in glass boxes and are handled by native assistants, who first place a forked stick over the neck, then grasp the snake firmly just behind the head and express the venom from the poison sacs. The antivenom is prepared by injecting horses, which are kept at Darjeeling where the climate is cooler, the venom being shipped to this place. It is claimed that the antivenom remains potent for a year at least, and, excepting moribund cases, 100 per cent. of efficiency is claimed. The commandant of the hospital was himself bitten by a cobra on the hospital grounds and recovered without serious symptoms. Cures have been effected even after injections made 48 hours after a bite.

No screens were seen in any hospital windows outside the jurisdiction of the U. S. In China, not a single queue was seen. The party was entertained at various points, a magnificent ball being held in Manila, and brass bands were in evidence at several places. The ship was met by an aviator in San Francisco Bay, who dropped an invitation from the Mayor for an automobile ride about the city and a luncheon.

---

THE WASSERMANN REACTION OF INFANTS AND CHILDREN. A CLINICAL STUDY. CHURCHILL—*Am. Jr. Child. Dis.* Vol. 13, No. 1.—Churchill had 102 cases picked at random in his hospital work, the age ranging from three days to 12 years. One hundred and eleven tests were made, of which twelve were of the spinal fluid. Thirty-nine gave positive reaction and 62 negative reaction, a positive reaction of 38 per cent. None of these cases entered the hospital with a diagnosis of spyphilis.

He collected his blood in a Wright capsule, using about 1 c. c. of blood. He found that in a small proportion of the cases that the spinal fluid will be negative when the blood is positive.

Various observers have shown that a positive test is obtained in from 96 to 100% of the manifest undoubted cases of syphilis in infants.

Knopfelmacher and Schwalbe had 29 cases of hyprocephalus and eight (27%) gave a positive Wassermann.

Dean in testing 330 cases in a hospital found a positive reaction in 15%, and of the 54 positive ones 44 were in idiots. He also showed that the number of positive cases decreased after 10 years of age.

Churchill's cases were entered with the following classification and gave positive Wassermanns:

| | | | | |
|---|---|---|---|---|
| Tuberculosis ........ | 26 | cases | 31% | positive |
| Nervous diseases...... | 16 | " | 52% | " |
| Circulatory diseases... | 16 | " | 43% | " |
| Respiratory diseases.. | 15 | " | 33% | " |
| Digestive diseases..... | 10 | " | 20% | " |
| Osseous diseases...... | 5 | " | 66% | " |
| Miscellaneous ....... | 13 | " | 45% | " |

Resume: 38% of 101 cases gave a positive reaction.

---

BOB VEAL. P. A. French of Cornell, *Am. Vet. Rev.*, 1912, No. 12, reports chemic and dietetic experiments tending to overthrow the popular prejudice. Immature flesh, generally, is relatively poor in fat and, hence, rich in water and poor in other solids, but there is neither theoretic nor practical reason to condem its use.

